# New Appliances and Things Medical

**Published:** 1895-09-28

**Authors:** 


### NEW APPLIANCES AND THINGS MEDICAL.

[We shall be glad to receive, at our Office, 428, Strand, London, W.C., from the manufacturers, specimens of all new preparations and appliances which may be brought out from time to time.]

### PETROLEUM JELLY.

(Snowdon and Sons, Millwall, London, E.)

Messrs. Snowdon and Sons have for many years devoted especial attention to the manufacture of petroleum jellies of both the yellow and white varieties, and have so far succeeded in their endeavour that their products are now recognised as among the very best which it is possible to obtain. The paraffinum molle of the English Pharmacopœia is defined as a semi solid mixture containing some of the softer or more fluid members of the paraffin series of hydrocarbons, and is met with in commerce under various fancy names. Among these, vaseline is perhaps the best known, and owing to this fact it has been proposed that the term vaselene should be used for all the paraffin hydrocarbons which have this semi-solid consistency. The petroleum jelly manufactured by Messrs. Snowdon and Sons conforms to all the requirements of the British Pharmacopœia, and is therefore a true vaselene. When our representative called on Messrs. Snowdon he obtained samples of their yellow and white jellies, and these samples have been submitted to our analyst for examination. He states that they are pure mineral jellies free from any trace of acid, alkalinity, or ash, and are free from any unpleasant odour or taste even when warmed. The white jelly has a slightly higher melting point and lower flash point than the yellow sample, but both are eminently suitable for use in manufacture of the various unguents when a neutral basis is required. We are pleased to find that an English firm can place such a good quality of petroleum jelly on the market, and can confidently recommend it for the many uses for which vaseline is usually prescribed.

